# Thrombin generation assays to personalize treatment in bleeding and thrombotic diseases

**DOI:** 10.3389/fcvm.2022.1033416

**Published:** 2022-11-10

**Authors:** Lars L. F. G. Valke, Sanna Rijpma, Danielle Meijer, Saskia E. M. Schols, Waander L. van Heerde

**Affiliations:** ^1^Department of Hematology, Radboud University Medical Center, Nijmegen, Netherlands; ^2^Hemophilia Treatment Center, Nijmegen, Netherlands; ^3^Department of Laboratory Medicine, Laboratory of Hematology, Radboud University Medical Center, Nijmegen, Netherlands; ^4^Enzyre BV, Novio Tech Campus, Nijmegen, Netherlands

**Keywords:** bleeding, personalized medicine, thrombin generation, thrombosis, hemophilia

## Abstract

Treatment of bleeding and thrombotic disorders is highly standardized and based on evidence-based medicine guidelines. These evidence-based treatment schemes are well accepted but may lead to either insufficient treatment or over-dosing, because the individuals’ hemostatic properties are not taken into account. This can potentially introduce bleeding or thrombotic complications in individual patients. With the incorporation of pharmacokinetic (PK) and pharmacodynamic (PK-PD) parameters, based on global assays such as thrombin generation assays (TGAs), a more personalized approach can be applied to treat either bleeding or thrombotic disorders. In this review, we will discuss the recent literature about the technical aspects of TGAs and the relation to diagnosis and management of bleeding and thrombotic disorders. In patients with bleeding disorders, such as hemophilia A or factor VII deficiency, TGAs can be used to identify patients with a more severe bleeding phenotype and also in the management with non-replacement therapy and/or bypassing therapy. These assays have also a role in patients with venous thrombo-embolism, but the usage of TGAs in patients with arterial thrombosis is less clear. However, there is a potential role for TGAs in the monitoring of (long-term) antithrombotic therapy, for example with the use of direct oral anticoagulants. Finally this review will discuss controversies, limitations and knowledge gaps in relation to the introduction of TGAs to personalize medicine in daily medical practice.

## Introduction

Hemostasis consists of a number of highly balanced processes to ensure blood flow and prevent unnecessary thrombosis and bleeding. A shift in this balance can lead to either of these conditions with associated morbidity and mortality, and an impairment in quality of life (1). In patients with thrombosis this balance has shifted to a state with increased activation of prothrombogenic factors while in patients with bleeding disorders it is associated with an inability to ensure sufficient stable platelet plug formation. Treatment of both disorders is highly standardized and is shaped according to evidence based medicine guidelines.

The most well-known coagulation related bleeding disorder, hemophilia A (HA), is associated with a high bleeding risk due to a deficiency of coagulation factor (F) VIII (2). Severe HA patients, who have a FVIII activity level of <1 IU/dL, are treated with prophylactic coagulation factor replacement therapy to prevent bleeding and subsequent joint damage (3). The schemes for prophylactic therapy are standardized and adjusted according to FVIII activity trough levels (4). However, some patients with adequate FVIII activity levels still experience bleeding symptoms (5). On the other hand, patients with thrombosis are treated with anticoagulant therapy, for example direct oral anticoagulants (DOACs) in venous thrombo-embolism (VTE) (6). The dosage of this therapy is based on large scale randomized controlled trials (RTCs) and it is effective to prevent recurrent thrombosis in most patients, without the introduction of bleeding complications. Nonetheless, some patients experience recurrent thrombosis despite adequate therapy compliance, while others experience life-threatening bleeding with the same therapeutic dosage (6). Therefore, despite current state-of-the-art evidence-based medicine, diagnosis and treatment of patients with hemostatic disorders is possibly suboptimal due to either insufficient treatment in one patient, while over-dosing in the other. Both introduces a risk for bleeding and thrombotic complications in the individual patient.

The assays to analyze patients with thrombotic and bleeding disorders consists of screening assays like the prothrombin time (PT) and activated partial thromboplastic time (APTT) and on confirmation assays of specific coagulation factors, like FVIII and protein C activity level determinations. Both kind of assays investigate a certain part of the coagulation cascade and do not take the intertwining processes into account. A global hemostasis assay can measure these multiple processes (7). Several global hemostasis assays exist (8), all with the idea to provide a more detailed impression of the individual patients hemostatic balance. The physician can use these parameters together with personal characteristics of the patient, like concomitant use of medication and comorbidities that interfere with coagulation, to provide a clinical applicable picture to eventually adapt therapy upon (9).

In this review we will discuss the recent literature about thrombin generation assays in relation to the management of bleeding and thrombotic disorders. Assays using whole blood or investigating fibrinolysis are beyond the scope of this review. For bleeding disorders, the main focus will lay on HA, as this is the most prevalent coagulation related bleeding disorder. For thrombotic disorders, the focus will lie on venous thrombo-embolic disorders and the treatment with DOACs, heparinoids and vitamin K-antagonists (VKA). Furthermore, we will discuss controversies, limitations and knowledge gaps in relation to the introduction of plasma-based global assays to personalize medicine in daily medical practice.

## Thrombin generation assays

The first reports of manual thrombin generation assays (TGAs) were published in 1953 (10, 11). Generation of thrombin is the result of effective activation of procoagulant factors of coagulation. Thrombin is a pivotal enzyme in hemostasis, as its generation represents a rate limiting step in fibrin formation, amongst its other key functions in hemostatic processes (12). It also functions as initiator of anticoagulant processes through potentiation of protein C upon binding of thrombin to thrombomodulin. *In vitro* measurement of thrombin generation uses substrates specific for thrombin cleaving activity that release a chromogenic or fluorogenic signal, to represent the net balance between these processes. Upon activation of the coagulation pathways through addition of tissue factor, phospholipids and calcium, the production of thrombin is initiated and accelerates exponentially, slows down, until it reaches a plateau phase (13). This can be measured in time, which gives insight in the net result of the hemostatic capacity.

**FIGURE 1:**
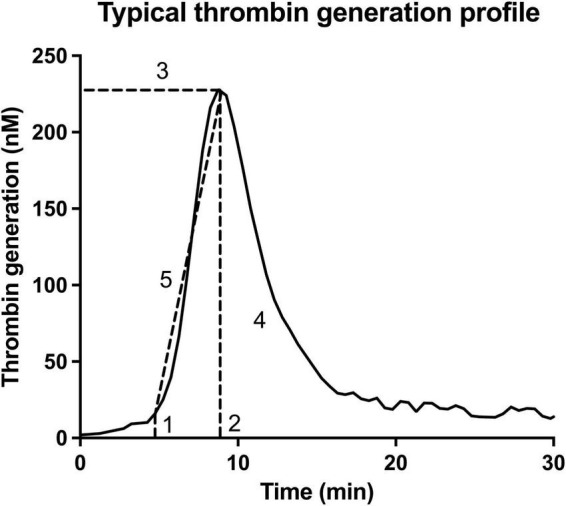
Essential parameters obtained with the thrombin generation assay. This thrombin generation assay is visually represented by the first derivative of the thrombin generation signal. The important parameters describing this curve include lag time (1), which describes the time between application of the trigger and the initiation of thrombin generation; time to thrombin peak (2) when the generation of thrombin is maximal; thrombin peak height (3) evaluating maximum generated quantity of thrombin; the area under the curve (AUC, also called endogenous thrombin potential (ETP), (4) where the total amount of thrombin generation is evaluated; as well as the velocity index (5) which describes the slope of the curve during the amplification phase.

The process of thrombin generation is visually represented by the first derivative of the thrombin generation signal as the signal accumulates in time, when measured with a chromogenic or fluorescent substrate (Figure 1). Important parameters describing this curve include lag time (1), which describes the time between application of the trigger and the initiation of thrombin generation, time to thrombin peak (2) when the generation of thrombin has reached its maximum, thrombin peak height (3) evaluating maximum generated quantity of thrombin, area under the curve (AUC, also called endogenous thrombin potential (ETP), (4) where the total amount of thrombin generation is evaluated, as well as the velocity index (5) which describes the slope of the curve during the amplification phase. These parameters vary in close conjunction; an increased thrombin peak height is mostly accompanied by an increased velocity index and ETP, while time to thrombin peak and also lag time are often shortened.

The lack of standardization of test parameters and composition of reagents has hampered comparability and harmonization of thrombin generation results to a great extent. First, the type of thrombin generation matrix varies from platelet poor plasma (PPP), platelet rich plasma (PRP) to whole blood. In most cases, PPP is used. For the TGA, standardization of preanalytical procedures to prepare PPP is essential. Comparison of different pre-analytical protocols where different blood collection systems, blood collection tubes, centrifugation method and time between collection and testing were evaluated, showed significant effects on thrombin generation results (14). Exemplifying centrifugation; a two-step centrifugation significantly decreased ETP potential compared to a one-step centrifugation. This is probably due to phospholipid contamination through microparticles or vesicles in samples that are centrifuged only once at a low centrifugation speed. Another factor that introduces pre-analytical variation is the venipuncture system and type of collection tube used. Addition of corn trypsin inhibitor (CTI) interferes with contact-activation, and thereby may reduce thrombin potential if contact activation occurs. However, this is also dependent on other factors like tissue factor concentration. The time and temperature at which a sample is preserved is another factor that can have a significant effect on thrombin generation results, as instability of coagulation factors does not allow prolonged storage times at room temperature. In conclusion, the establishment of a standardized pre-analytical protocol will aid significantly in harmonizing TGAs. The International Society on Thrombosis and Haemostasis (ISTH) Scientific and Standardization Subcommittee (SSC) suggested specific preanalytical conditions for the measurement of thrombin generation for the indication of hemophilia, which are described in ref (15).

Composition of the method that is applied to produce a fluorescent signal, is another important factor that hampers harmonization of thrombin generation results. Two main specific thrombin substrates have been applied to measure its generation, either attached to a chromogenic or fluorogenic signal, para-nitroaniline (pNA) and 7-amino-4-methylcoumar (AMC), respectively (16, 17). In both methods, the caged signal molecule is released by thrombin mediated proteolysis. The peptide is specific for thrombin and has a relative high Km and low Vmax to avoid substrate depletion. The chromogenic pNA signal is quantified through extinction measurement at 405 nM. Defibrination or inhibition of fibrin polymerization is required for this assay due to interference of the chromogenic signal during clot formation. Fluorescent AMC signal is not sensitive toward interference through clot formation and is excitated at a wavelength of 390 nm resulting in a peak at an emission wavelength of 460 nm. The rate at which these signal molecules are released varies between chromogenic and fluorescent substrates, and also the amino acid composition of the tri-peptide, which hampers comparison of results for these two methods (8).

Calibration of the assay can be performed by measurement of a known range of thrombin concentrations, or thrombin bound to alpha-2-macroglobulin (α2M). Fluorescent substrates in the TGA are also cleaved by thrombin complexed to α2M, whereas thrombin complexed to α2M is not biologically active in fibrinogen activation (18). Moreover, in specific patients, such as the pediatric population and neonates, the α2M concentrations can be significantly altered compared to the adult population (19). The biological active free thrombin component can therefore differ from the measured total thrombin activity. In some assays, the thrombin generation curve is corrected for the amount of thrombin that is bound to α2M, while in other assays no correction for α2M is applied/necessary (20).

Furthermore, correction for quenching of the fluorescent signal differs between methods. Correcting for quenching, or the inner filter effect, of the fluorescent signal in patient samples with varying composition is a non-linear phenomenon and may lead to an underestimation of thrombin generation and increased variation in test results (21). Some methods correct for quenching by the application of mathematical correction using an intra-assay control sample, in other methods no correction for quenching is applied (22).

Thrombin generation is initiated by the addition of tissue factor and phospholipids, which will activate the extrinsic pathway and stimulate coagulation, respectively. The concentration of these trigger components can differ and will alter sensitivity of the assay toward different types of coagulation disorders. Reagent compositions applying low concentration of tissue factor and phospholipids are most sensitive toward bleeding disorders (23), whereas a higher tissue factor concentration can be predictive for a prothrombotic phenotype (24). In the anticoagulated patient, higher concentrations of tissue factor and phospholipids are suggested to be more appropriate (25). Tissue factor can be derived from either tissue (human plasma, human placenta, and rabbit), or through recombinant expression. Apart from the concentrations in trigger reagent, also the composition of the applied phospholipids (e.g., percentage of phosphatidylserine, phosphatidylcholine, phosphatidylethanolamine) and the size distribution of the vesicles containing these phospholipids may vary. Imprecision in thrombin generation results is increased using different sources of tissue factor and phospholipids, especially at low concentrations (26). For most commercial available reagents, exact composition for the different reagents is not disclosed, and variation between batches could be expected (25).

Normalization of the obtained thrombin generation results, as suggested by the ISTH-SSC, is often advocated to reduce inter-assay and inter-laboratory variation, and to aid in the interpretation of the results of the different thrombin generation parameters (15, 26). However, determination and application of a normal sample is complex because no reference material exists, different pool samples, or commercially available control samples have varying composition and therefore may increase variation between assays as well as hamper comparability and interpretation of results of different assays.

Finally, thrombin generation can be determined with a manual, semi-automated or automated assay. The automated assays that are currently available for the evaluation of thrombin generation apply fluorescent substrates, but reagent composition, and calibration and mathematical procedures differ (8). The (semi-)automated assays that are often applied are the Calibrated Automated Thrombography (CAT, Stago), ST Genesia (Stago), and Ceveron TGA (Technoclone). A comparison of thrombin generation performance of these assays for patient populations with specific bleeding or thrombotic phenotypes, as well as patients treated with anticoagulants, has not been reported. Therefore, the effect of differences in reagent composition, calibration, correction methods and normalization on thrombin generation results cannot be interpreted for these patient population when different assays are used.

## Bleeding disorders

TGAs are used in the analysis of bleeding disorders, to give an impression of the clinical phenotype of patients with bleeding disorders and to monitor treatment (for summary of most important findings, see Table 1). These different applications will be discussed here.

**TABLE 1:** Summary of thrombin generation assay characteristics and findings in bleeding and thrombotic diseases.

Clinical scenario	TGA conditions*	TGA parameter	Important findings	References
**Bleeding disorders**				
Diagnosis of bleeding of unknown cause	PPP, low	Lag time, TPH, TP	Lower thrombin generation in patients with bleeding of unknown cause compared to normal controls.	(31, 33–36)
Clinical phenotype of bleeding disorders	PPP, low PRP, low	TPH, TP TPH, TP	HA patients with severe bleeding have lower thrombin generation despite equal FVIII activity level; thrombin generation is lower in RBD patients with bleeding. FXI deficiency bleeding phenotype can be distinguished with high diagnostic accuracy.	(40–44, 49)
**Treatment monitoring**				
Factor concentrate	PPP, low	TPH, TP	Bleeding patients with prophylactic therapy have lower thrombin generation than not bleeding patients; thrombin generation is suggested to be used for individualized prophylactic treatment schemes.	(55–66)
Bypassing agents (BPA)	PPP, low	TPH, TP	Monitoring of the effect of BPAs on hemostasis and selecting the bypassing agent with the most efficacy in the individual patient.	(69–77)
Emicizumab	PPP, low	TPH, TP	Emicizumab restores thrombin generation to 20-30% of normal; possibility to monitor the effect of combination therapy with emicizumab and FVIII concentrate or BPAs.	(84–93)
Anti-TFPI	PPP, low	TPH, TP	Patients reaching the prespecified concentration of concizumab had normalization of thrombin generation; possibility to monitor concomitant use of FVIII concentrate or BPAs.	(99–102)
Antithrombin antisense	PPP, low	TPH, TP	Normalization of thrombin generation when antithrombin was > 75% reduced; monitoring of combined therapies.	(80, 81, 103)
**Thrombotic disorders**				
Diagnosis of first and recurrent VTE	PPP, high	TPH, TP	Higher rates of first and recurrent thrombosis in patients with increased thrombin generation; lower risk of recurrent thrombosis with low thrombin generation.	(105–114)
Diagnosis of arterial thrombosis	PRP, high	Lag time, TPH, TP	Some indications that thrombin generation is increased in coronary artery disease, for ischemic stroke conflicting evidence.	(118–132)
Antiphospholipid syndrome	PPP, high	Lag time, TPH; TP	Normalized thrombin peak height/lag time ratio can identify patients with antiphospholipid syndrome; identification of patients with suboptimal treatment according to thrombin generation despite adequate INR.	(135, 137–146)
**Anti-thrombotic therapy**				
Heparinoids	Variable, high	TPH, TP	Tinzaparin provides greater thrombin generation reduction compared to other heparinoids; anti-Xa measurement possibly less indicative for hypercoagulable state.	(150–155)
Vitamin K antagonists (VKA)	PPP, high	Lag time, TPH, TP	INR has a correlation with lag time; lower thrombin generation is associated with bleeding in VKA treated patients.	(156–159)
DOACs	PPP, high	TPH, PT	Thrombin generation is already inhibited at a low DOAC concentration; reversal with BPAs can be monitored with thrombin generation; low correlation between anti-Xa concentration and thrombin generation.	(162–180)
Antiplatelet therapy	Variable#	Velocity of thrombin generation, TPH, PT	PPP is less sensitive for the measurement of thrombin generation then PRP; velocity of thrombin generation, TPH and TP are most affected by antiplatelet therapy.	(181–184)

*Thrombin generation assay characteristics that give the best results in this condition or are used by most studies in this area. TGA determined in platelet poor plasma (PPP) or platelet rich plasma (PRP), with low (usually 1 pM) or high (usually 5 pM) tissue factor as starting reagent.

^#^In studies reporting TGAs with antiplatelet therapy PPP and PRP are used, with both low and high concentrations of tissue factor. BPA, bypassing agent; DOACs, direct oral anticoagulant; FVIII, factor VIII; FXI, factor XI; INR, international normalized ratio; PPP, platelet poor plasma; PRP, platelet rich plasma; RBD, rare bleeding disorder; TFPI, tissue factor pathway inhibitor; TGA, thrombin generation assay; TP, thrombin potential; TPH, thrombin peak height; VTE, venous thrombo-embolism; VKA, vitamin K antagonist. Lars L. F. G. Valke: LV Sanna Rijpma: SR Danielle Meijer: DM Saskia E. M. Schols: SS Waander L. van Heerde: WH.

### Diagnosis of bleeding disorders

Patients with a mild to moderate bleeding tendency are subjected to multiple diagnostic assays to detect the hemostatic abnormality (27). However, in one in three patients a definitive diagnosis cannot be established (28), a condition called “bleeding of unknown cause” (BUC) (29). Several research groups have investigated the additional value of thrombin generation measured in PPP in patients with BUC (30–36). These studies provided conflicting results, with two, older studies showing no association (30, 32), while other studies found abnormalities in thrombin generation parameters (31, 33–36). The studies that found decreased thrombin generation were generally larger than the studies that did not, and used lower TF concentrations to start the TGA. As a hypocoagulable state can best be detected with a low TF concentration, it is possible that the amount of TF can explain this difference. The most prevalent described abnormalities were a prolonged lag time (31, 33–36), decreased thrombin peak height (31, 33), and decreased ETP (31, 33, 34, 36). However, the main limitation of TGAs in BUC patients is the overlap in thrombin generation results between patients with BUC and healthy controls. Therefore, reference ranges for diagnosis of bleeding based on decreased thrombin generation or increased lag time need to be determined. A specific disease entity (for example, bleeding based on impaired thrombin generation, as data from previous mentioned studies imply) could improve scientific research and treatment for these patients.

### Difference in clinical phenotype of bleeding disorders

The value of TGAs is best researched in patients with HA, where it shows an association with clinical bleeding phenotype. Only a few papers report about TGA and bleeding tendency in patients with rare bleeding disorders (RBDs) (37–39).

Hemophilia patients with the same genotypic variant and factor coagulation activity level often show differences in clinical bleeding phenotype, possibly due to alterations in other coagulation factors than FVIII (in HA) and FIX (in hemophilia B; HB). This hypothesis was confirmed by multiple small studies in which the bleeding phenotype correlated with one or multiple TGA parameters in PPP (40–44). One study was unable to establish this association (45), possible due to the use of the Tosetto bleeding score, that is developed for Von Willebrand disease (VWD), instead of a hemophilia specific bleeding score (46). Another study investigated the relationship between FVIII activity level, genetic variations and inhibitor status. Patients with an inhibitor, a neutralizing antibody against FVIII making replacement therapy ineffective, showed decreased thrombin generation capacity compared to patients without inhibitors, despite equal FVIII activity levels (47). Thereby it can be concluded that TGAs can play a role in unraveling the clinical bleeding phenotype of hemophilia patients and even may play a role in how to treat these patients.

It is known that in patients with FXI deficiency (also known as hemophilia C) FXI activity level and bleeding phenotype do not correlate with each other (48). However, thrombin generation could possibly distinguish patients with different FXI activity levels and patients with and without bleeding. It appeared that certain sampling and testing conditions influenced the results of thrombin generation. In PPP with CTI, thrombin generation did not identify FXI deficient patients from normal controls. But when PRP with CTI and low TF was used, it could differentiate between patients with and without FXI deficiency. Furthermore, differences in thrombin peak height and thrombin potential had a high diagnostic accuracy for identifying bleeding from non-bleeding patients (49).

RBDs are a heterogenous group of diseases with different coagulation factor defects. All these diseases have a variable bleeding tendency, that only partially can be explained by the activity level of the missing or depleted coagulation factor. A retrospective study of RBD patients showed that thrombin peak height and ETP measured in PPP were significant lower in patients with major bleeding, compared to patients with minor bleeding (38). Major bleeding patients had ETP values <20% of normal (38). This was comparable to the results of another study that found that all patients with major bleeding had an ETP of <20% of normal, while RBD patients with an ETP >30% of normal had no clinically relevant bleeding symptoms (37). The added value of TGAs in patients with RBDs was confirmed in a third study which showed that it could better predict significant bleeding compared to factor activity level (39).

Lastly, a study involving patients with von Willebrand disease (VWD) showed that patients with a mild bleeding phenotype had higher thrombin peak height compared to patients with more severe bleeding. The thrombin peak height and velocity of thrombin generation both correlated with VWF activity level and FVIII activity levels. This was observed in both PRP as well as PPP. Plasma FVIII activity level was the main driver of thrombin generation in this study (50).

### Treatment of bleeding disorders

#### Factor concentrate

The use of TGAs is mainly investigated in the treatment of HA patients. Spiking studies were first reported, in which plasma of HA patients was spiked with multiple concentrations of FVIII and thrombin generation in PPP was measured. Multiple *in vitro* spiking studies showed that thrombin generation reaches a plateau phase when FVIII activity level is between 20 and 50 IU/dL (40, 51–53), with only one study failed to show this plateau phase and suggested a linear correlation between FVIII activity level and thrombin generation (54). All studies reported major variation in the FVIII activity level at which thrombin generation was normalized in individual patients, indicating a need to individualize FVIII replacement therapy dosage to obtain normal thrombin generation in the TGA.

The individual response to FVIII replacement therapy was also reported in multiple *in vivo* studies with HA patients. A strong correlation between FVIII activity level and thrombin generation parameters (except lag time) in PPP was found (55–61), but the inter-patient variation of thrombin generation was large after a standard infusion of FVIII. For example, some patients generate normal total amounts of thrombin with subtherapeutic FVIII activity levels while others don’t show normalization of thrombin generation despite adequate FVIII activity levels (56). On the other hand, the intra-patient variation was small, thereby suggesting that the thrombin generation in an individual patients is predictable (57, 60).

Furthermore, multiple studies showed that the thrombin generation response had a prolonged duration after a single bolus of factor VIII concentrate compared to FVIII activity level. FVIII activity level declined over time after administration of FVIII replacement therapy, while thrombin generation remained elevated (55, 57, 60, 62). This effect was, again, variable between patients, implicating that other factors, like very low-titer inhibitors or level of other coagulation factors could play a role. This was further investigated in a pharmacokinetic (PK)/pharmacodynamic (PD) modeling study which showed that on average a 50% ETP level (EC_50_) was reached with only 11.6 IU/dL FVIII activity level increase. However, the inter-individual differences were large, underscoring the existence of an individual unique thrombin generation profile. For example, three patients with similar PK-profiles exhibited EC_50_ values that varied from 7.9 to 29.8 IU/dL FVIII (63).

A second PK/PD-modeling study also incorporated bleeding in their analysis. This study was based on the data of the GENA-21 study, which already had shown that FVIII activity level did not correlate with bleeding symptoms during prophylactic FVIII replacement therapy (64). However, patients with bleeding had significant lower thrombin generation compared to patients who had no bleeding symptoms (65). In this PK/PD-modeling study the authors found that patients with the highest ETP at baseline, had the lowest bleeding rate even with the lowest FVIII replacement therapy dosage compared to patients with the lowest ETP at baseline and highest FVIII replacement therapy dosage (66). This study showed that individualized dosing of FVIII replacement therapy based on ETP is superior in bleeding outcomes with this specific FVIII product.

In patients with hemophilia B (HB) a PK-PD model study was performed with a recombinant FIX-Fc fusion protein (eftrenonacog-alfa) and showed that velocity of thrombin generation showed the best correlation with FIX activity level. Thrombin peak height and ETP were the following parameters that decreased over time after replacement therapy. However, bleeding was not assessed in this study (67).

Lastly, a cross-over study assessed the PK-PD relationship between supplementation of plasma derived (pd) FVII and recombinant activated FVII (rFVIIa) in patients with FVII deficiency. This study identified lag time as the best effect-response parameter. In the PD-analysis, it was shown that the EC_50_ was only 2 IU/dL FVII activity for both pdFVII and rFVIIa. Furthermore, they showed that a plasma FVII activity level of 3-4 IU/dL was sufficient to reach lag time values comparable with the upper limit of healthy controls (68). These data underscore the discriminating value of thrombin generation in RBDs, especially if supplementation therapy is difficult to monitor because of long turn-around times for certain coagulation factor activity level determinations or because of replacement with rFVIIa, activated prothrombin complex concentrate (aPCC) or plasma (see section “Bypassing agents”).

#### Bypassing agents

Thrombin generation assays have an additional value in the monitoring of bypassing agents (BPAs), like rFVIIa and aPCC, in hemophilia patients. These products are mainly used in patients with inhibitors because FVIII replacement therapy is ineffective. Since BPAs cannot be monitored with a single factor assay, especially if it is given in combination with other treatment modalities, performing thrombin generation is an attractive alternative (9).

Activated prothrombin complex concentrates are shown to restore thrombin generation by spiking plasma samples of HA patients with 1–2 IU/mL aPCC (which corresponds with the therapeutic dose of 50–100 IU/kg) (69–71). In a PK/PD-study with aPCC in three HA patients, thrombin generation was restored after administration of 65–100 IU/kg aPCC and it diminished to 50% between 4 and 7 h (71). In pediatric HA patients with inhibitors, thrombin generation was restored to 80% of normal at peak aPCC levels after administration of 60–100 IU/kg. Thrombin potential remained enhanced 2.6 fold at trough aPCC level compared to control inhibitor plasma, indicating longer lasting effects on thrombin generation (72).

The effect of rFVIIa on thrombin generation was found to be less than aPCC in a cross-over study (73). The thrombin generation response after a bolus rFVIIa was highest after 30–60 min and decreased over a period of 4 h, anticipating the half-life of rFVIIa (74). All studies investigating the effect of rFVIIa in HA patients have shown that thrombin peak height and ETP are increased in PPP, but do not reach normal values (75, 76). Furthermore, individual patients show a difference in thrombin generation response to rFVIIa, with some patients having a poor response (76).

Since it is impossible to predict the hemostatic response of an individual patient to BPA therapy, Dargaud et al. proposed a three step model to individually tailor therapy. They investigated the performance of this model in six HA patients during ten invasive procedures. No bleeding occurred in the patients in whom ETP was normalized with the selected therapy (77).

#### Non-factor replacement therapy

During recent years, non-factor replacement therapies have been introduced in the treatment landscape of HA. Emicizumab, a bispecific monoclonal antibody that forms a pseudo-tenase complex (78), is the first non-factor replacement drug receiving market authorization, with other treatments like anti-tissue factor pathway inhibitor (anti-TFPI, e.g., concizumab) (79) and a mRNA against antithrombin (fitusiran) (80, 81) following pursuit. Because the hemostatic effects of these non-factor replacement therapies cannot be monitored with conventional assays, or lead to falsely normalized FVIII activity level (82), it is better to assess the end-product of hemostasis with TGAs, especially if used in combination with other treatment modalities (83).

The first study of emicizumab in FVIII depleted plasma showed thrombin generation parameters increasing to half of normal (82). One study found a linear correlation between emicizumab concentration and thrombin peak height measured in PPP (84). Moreover, results showed that thrombin potential in emicizumab treated HA patients reached a plateau at 20–30% of normal (84), which was replicated in other studies (85, 86). Moreover, it was shown that thrombin generation was lower in infants younger than 1 year, compared to older children and adults, possibly due to a faster clearance of emicizumab (85, 87, 88). Finally, thrombin peak height and ETP were significantly lower in patients who presented with major bleedings (85, 89).

TGAs can mainly be used to monitor combined treatment modalities, in which measurement of individual components of therapy is impossible. Because emicizumab was first investigated in HA patients with inhibitors, most data exist about the combination of emicizumab with BPAs. However, one *in vitro* spiking study showed that combination therapy of emicizumab with plasma derived FVIII/VWF (pdFVIII/VWF) did not increase thrombin generation above levels observed in PPP with monotherapy pdFVIII/VWF. This is expected because FVIIIa has a greater affinity for the tenase complex than emicizumab (90). Moreover, in multiple spiking studies (90–92), it was observed that combination therapy of emicizumab with aPCC in low dosage (5 IU/kg) already normalized thrombin generation. APCC in higher dosage (>30 IU/kg) increase thrombin generation above normal values, to even more than eight-fold normal values with a dosage of 100 IU/kg (90–92). On the other hand, when HA plasma with emicizumab was spiked with rFVIIa in the highest dosage of 270 μg/kg, thrombin generation did not exceed normal values (90–92). Most importantly, it was discovered that activated FIX, in aPCC was responsible for the synergistic effect of emicizumab and aPCC *in vitro* (93). Whether this also occurs *in vivo* is still in debate. It can be concluded, however, that HA patients treated with emicizumab who need additional therapies should be treated with care and thrombin generation should be closely monitored. Patients without inhibitors can safely be treated with FVIII replacement therapy, because activated FVIII has a greater affinity for FIXa and FX than emicizumab and a synergistic effect is not expected (83, 94). However, patients with inhibitors can be treated with rFVIIa in normal dosage, and if not available or not effective, with very low dose aPCC with careful thrombin generation monitoring as multiple patients have developed thrombosis after administration of aPCC (dosed >200 IU/kg/day) in combination with emicizumab (78).

Anti-TFPI treatment enhances the initiation phase of coagulation by inhibiting the shutdown effect of TFPI resulting in a prolonged TF-FVIIa activity, leading to an increased activation of FX and eventually thrombin (95). The now discontinued agent BAX-499 already showed improved thrombin generation in hemophilic plasma (96). Additionally, spiking studies with two different anti-TFPI antibodies (marstacimab and befovacimab) both increased thrombin generation to a level that was approximately equal to a FVIII activity level of 40% (97, 98). Most pharmacodynamic studies are performed with concizumab which showed a dose-dependent increase in thrombin generation, even in plasma of healthy volunteers (79, 99). Afterward, pharmacodynamic monitoring was used to determine the eventually investigated dose of concizumab (100). Patients who reached the prespecified concentration of concizumab (>100 ng/ml) showed normalization of thrombin generation (101). Lastly, concomitant therapy of concizumab with aPCC, rFVIIa, and FVIII showed additive effects, instead of exponentially effects such as between aPCC and emicizumab (102). Therefore, concizumab can be combined with other treatment modalities, but dosages should be adjusted and monitored with TGAs to provide safe and effective therapy.

Antithrombin lowering can be established with fitusiran, an anti-sense oligonucleotide directed against antithrombin mRNA, leading to decreased inhibition of coagulation. Studies showed increasing amounts of thrombin generation with further reducing antithrombin with reaching near normal levels of thrombin generation when antithrombin was >75% reduced (80, 81). Comparable with concizumab, fitusiran combined with aPCC or rFVIIa had additive effects on thrombin generation (103).

## Thrombosis

The TGA can be used in the analysis of patients with thrombosis, for example to analyze the prothrombogenic phenotype in patients with (recurrent) VTE (104), and to monitor treatment with anticoagulant therapy (for most important findings, see Table 1).

### Analysis of thrombotic tendency

#### First and recurrent venous thrombo-embolism

Venous thrombo-embolism consists of pulmonary embolism and deep vein thrombosis and is common in the general population. Patients are treated with anticoagulants to prevent further progression of the thrombus and preventing recurrent thrombosis. However, the ideal duration of anticoagulation in the individual patient is unknown and decisions about stopping/continuing anticoagulation were made on clinical characteristics and patient preferences. Possibly, the TGA could help indicate which patient has a hypercoagulable phenotype and has a high risk for first or recurrent VTE. This is investigated in multiple studies (105–114). The first indication that thrombin generation could influence VTE recurrence was with a RCT in which D-dimer level measured one month after discontinuation was used to indicate prolonged anticoagulation. Patients with an elevated D-dimer level who restarted anticoagulation had a significant lower chance of recurrent VTE than patients without anticoagulation (115). Furthermore, it was shown that patients with VTE have significantly higher ETP values than controls without VTE (105, 106, 109). One study found that thrombin generation was higher in individuals with an additional risk factor for the development of VTE than patients with an idiopathic VTE (105). Another study, however, reported higher thrombin generation in patients with idiopathic VTE compared to those with provoked VTE, even after correction for FVIII and D-dimer levels (112). Furthermore, multiple studies showed that addition of thrombomodulin to the TGA was able to magnify the differences found between patients and controls (105–107).

The risk of recurrent VTE can be estimated with TGA with increased hazard ratios (HR) ranging from 1.6 to 4.0 for increased ETP and subsequent recurrent VTE (107, 108, 113). The HR for recurrent VTE based on thrombin peak height was even 4.6 in one study (107). One study could not establish an increased risk for recurrent VTE (HR 1.1) when elevated ETP was used to distinguish patients and controls (109). This different conclusion can be explained by the study design, as the last study was a case-control study, while all others were prospective cohort studies. Interestingly, a cohort study also showed that patients with low thrombin generation had a lower risk of recurrent VTE (HR 0.40) (111). Thereby confirming the risk association between thrombin generation and the risk of recurrent VTE.

Lastly, patients with cancer have a high risk of VTE development. Ay et al. performed TGA in 1033 patients with various types of solid tumors and found a HR of 2.1 for the development of a VTE event in patients with the highest quartile of thrombin generation. Incidence of VTE in the first 6 months was 11% in this quartile, compared to 4% in patients with lower thrombin peak height (116). Therefore, it can be concluded that thrombin generation might be a useful tool to predict first and recurrent VTE incidence, in patients with idiopathic and provoked VTE, and in patients with a malignancy. However, it should be noted that absolute cut-off values of thrombin generations parameters are not possible, because a large overlap in thrombin generation profiles exists between VTE patients (114).

#### Arterial thrombosis

In contrast to VTE, the role of thrombin generation measurement is less clear in patients with arterial thrombosis. Arterial thrombosis is a leading cause of death worldwide and consists of coronary artery disease (CAD) and ischemic stroke. It is a complex interaction between the long lasting process of atherosclerosis of the main arteries, in combination with acute rupture of an atherosclerotic plaque that provokes thrombus formation at the site of injury. Only if the thrombus limits blood flow to the affected organ, symptoms can be reported by the patient. Since atherosclerosis and inflammation are strongly linked to each other and inflammation has a role in thrombin generation, the exact relationship between arterial thrombosis and thrombin generation is hard to establish and conflicting evidence is reported (117).

It is shown that patients with CAD have higher thrombin generation during an acute myocardial infarction and during the chronic phase, compared to patients with stable disease (118). This suggests that these patients are in a more hypercoagulable state and are more prone to arterial thrombosis (119). Increased thrombin generation parameters (thrombin potential and thrombin peak height) are often described in patients with acute MI or CAD (118, 120–122), but also a prolonged lag time is described (123). However, other studies describe a more U-shaped association between thrombin potential and CAD (124, 125). The association between enhanced thrombin generation and arterial thrombosis was further investigated in a case-control study with patients with an in-stent thrombosis after myocardial infarction. Here again, it showed that patients had higher thrombin generation compared to controls who did not have in-stent thrombosis (126). Furthermore, patients with residual detectable thrombin generation after percutaneous coronary intervention (PCI) despite optimal antiplatelet and periprocedural anticoagulant therapy had a higher risk of cardiovascular death (127).

For ischemic stroke, evidence is less clear. In one study, young stroke patients had an increased thrombin potential in PRP, while the association was not found in PPP (128). Multiple, smaller studies did not show an association between thrombin generation and adverse events (129, 130). These studies could be hampered by their sample size, since in one cohort study of more than 9,000 persons, a significant association was found between thrombin generation and the development of ischemic stroke. This study suggests that ischemic stroke could be prevented by diminishing the hypercoagulable state in these patients (122). On the other hand, another prospective cohort study found a significant inverse relationship between thrombin generation and the development of stroke (121).

In summary, the relationship between thrombin generation and arterial thrombosis is not readily defined. Thrombin generation in patients with CAD is increased in most studies, but the effect is only substantial. In patients with ischemic stroke, the evidence is even less clear-cut. These differences can be explained by study design or study population (117). Furthermore, the influence of traditional risk factors for cardiovascular disease on thrombin generation cannot be excluded. For example, obesity has been shown to increase thrombin potential (131). Also, another study showed that the concentration of apolipoprotein C-III was an independent risk factor for CAD, but also that it was associated with thrombin peak height and thrombin potential (132). Still, it is established that higher thrombin potential is associated with increased total mortality (131). Further research in this field should elaborate on thrombin generation in both PPP and PRP, because thrombin generation in arterial thrombosis is an interplay between vessel wall, platelets and coagulation factors.

#### Antiphospholipid syndrome

The antiphospholipid syndrome (APS) is characterized by obstetric complications and/or arterial/venous thrombosis in combination with typical antiphospholipid antibodies (aPL antibodies: lupus anticoagulant (LAC) and/or anti-β2 glycoprotein I (aβ2GPI) and/or anti-cardiolipin (AC)) measured twice with at least 12 weeks in between (133). Major assay heterogeneity and lack of standardization cause problems with the diagnosis of APS (134). Also, non-pathogenic aPL antibodies can be encountered, for example in the presence of certain infections or medication. Furthermore, not all carriers of aPL antibodies develop thrombo-embolic complications. In this regard, the TGA may play an important distinguishable role (134).

In the diagnostic process, a chromogenic TGA was able to detecting all three aPL antibodies and could even distinguish between APS antibodies and antibodies arisen from transient causes, such as infections. However, this assay used purified antibodies and NPP, making it not available for use in routine clinical practice (135). Multiple groups have shown that aPL antibodies cause a lag time prolongation in the TGA, potentially due to shielding of the exogenous added phospholipids (136, 137). However, in these patients, thrombin peak height was increased as well, which led to the proposition of the use of the normalized peak height/lag time ratio (PH/LT-ratio) (138). This ratio was able to detect LAC antibodies with high sensitivity, even in anticoagulated patients (139). But additional research is necessary to establish that increased thrombin generation is due to APS instead of other causes, since increased thrombin generation is also seen in patients with VTE (as described in section “First and recurrent venous thrombo-embolism”).

Multiple studies have shown that the increased thrombin generation observed in patients with APS is mainly due to increased activated protein C (APC) resistance (137, 140, 141). This APC resistance was associated with thrombotic events (139–143), and was even incorporated in a ratio that could predict thrombosis over time (144). Lastly, TGAs can also be used to determine the degree of anticoagulation in patients with APS (145, 146). It even showed that a subgroup of patients had increased thrombin generation despite adequate international normalized ratio (INR) values. Thereby it is a possible tool to identity APS patients with an ongoing prothrombotic state despite therapy with vitamin K-antagonists (VKAs) (145). In the next paragraph, anticoagulation monitoring with TGAs will be further described. Thereby, it can be concluded that TGAs can be used in combination with classic APS assays to provide a more detailed impression of the hypercoagulable state of patients with thrombosis due to APS. Furthermore, monitoring of anticoagulation in these patients can be helpful, as recurrent thrombosis is common in APS patients (147).

### Treatment with anticoagulant therapy

Arterial and venous thrombo-embolic disorders are treated with different kinds of anticoagulant therapies, depending on the indication and patient characteristics. For most of these treatments, some kind of test to monitor the effect exists in the laboratory. However, mostly this encompasses a part of the coagulation cascade, like anti-Xa monitoring for heparinoids or LMWH, and it does not take hyper- or hypocoagulability of the patient into account (148). This part of the review will focus on anticoagulant therapy with heparinoids, VKA, DOACs and lastly antiplatelet therapy.

#### Heparinoids

Heparin treatment can be divided in unfractionated heparin (UFH) and low molecular weight heparin (LMWH) therapy. UFH treatment needs to be monitored by measurement of APTT and/or anti-Xa, while treatment with LMWH is often fixed-dosed or weight-based dosed (149). However, LMWH is sometimes monitored with anti-Xa determination at the extremes of the weight spectrum (e.g., cachexia and morbid obesity) and in patients suffering from renal insufficiency. With anti-Xa monitoring, it appears that some patients show widely different anti-Xa activity levels with the same dosage, therefore, thrombin generation monitoring could be of interest in patients treated with UFH or LMWH (149).

The anticoagulant effect of UFH is comparable with different kinds of LMWH in spiked PRP (150). This study showed that tinzaparin had greater thrombin generation inhibitory effects compared to UFH and other LMWHs at the same anti-Xa activity level (150), which was confirmed in a second study that compared enoxaparin with tinzaparin (151). Moreover, it was shown that fondaparinux, a synthetic pentasaccharide which inhibits Xa formation via antithrombin, had less inhibitory effect on thrombin generation if compared to LMWH (150, 152).

Thrombin generation in the presence of LMWH was also measured in some specific populations. It is known that thrombin generation increases during pregnancy. In one study, healthy pregnant women, pregnant women with mild (e.g., heterozygous factor V Leiden) and severe thrombophilia (e.g., homozygous factor V Leiden) were followed each trimester with thrombin generation measurement. In women with severe thrombophilia, thrombin generation increased more than in women without thrombophilia (153). Prophylactic LMWH dosage inhibited thrombin generation. However, in the third trimester, thrombin generation was significantly elevated despite stable anti-Xa activity levels over time (153). Suggesting that pregnant women are in a hypercoagulable state despite fixed prophylactic LMWH therapy. This effect was also shown in morbidly obese pregnant women, which showed higher thrombin generation parameters compared to normal weight pregnant women. Interestingly, the authors showed that a weight-based prophylactic LMWH dosage led to significant lower ETP values compared to standard-dosed LMWH (154). Lastly, the TGA was able to detect a hypercoagulable state in patients with cancer and showed normalization of thrombin generation whilst patients were on LMWH therapy (155).

#### Vitamin K antagonists

Before the introduction of DOACs, VKA were the main oral anticoagulants used. Dosing of VKA was personalized by measurement of the INR with subsequent dosage adjustments because multiple factors, like diet and genetic variants, influence the effect of VKA. Bleeding is the main risk of anticoagulation, therefore the goal is to keep the INR in a prespecified range. However, the INR only gives an impression of procoagulant factors, while anticoagulant factors are also influenced by VKAs. Therefore it would be interesting to know if VKAs could also be monitored with TGA.

Thrombin generation in the VKA treated patient showed a significant correlation with INR values, especially for lag time (156). In another study, however, some patients showed persisting thrombin generation despite adequate INR values (145), possibly indicating that they were still prone to recurrent VTE. When warfarin was compared with rivaroxaban, a DOAC, it appeared that overall thrombin generation parameters were comparable. Rivaroxaban exhibited slightly longer lag time, time to thrombin peak and lower thrombin peak height, while warfarin showed a lower ETP (157). However, in a study investigating an APS patient, rivaroxaban showed higher thrombin generation compared to warfarin and enoxaparin (146).

Interestingly, in a prospective study investigating bleeding episodes in patients using VKAs, it appeared that patients with bleeding had significant lower thrombin peak height and ETP values measured with whole blood TGA, compared to patients who did not bleed. The patients with bleeding also had higher HAS-BLED scores, indicating that both whole blood TGA and HAS-BLED score showed an association with bleeding (158). In another prospective cross-sectional study ETP was lower in warfarin treated patients who presented at the emergency department with bleeding, compared to warfarin treated patients who presented with another medical emergency, while INR was within target range in both groups (159).

#### Direct oral anticoagulants

Direct oral anticoagulants can inhibit thrombin (dabigatran) or FXa (apixaban, edoxaban, and rivaroxaban) and are given in a fixed dosing regimen, either once daily (edoxaban and rivaroxaban) or twice daily (apixaban and dabigatran). The main advantage of DOACs over VKAs is that monitoring of anticoagulation is not required (160). However, in some instances, for example in case of bleeding, recurrent thrombosis, or renal insufficiency, monitoring the effect of anticoagulation with DOACs can be of interest. In this regard, the anti-IIa or anti-Xa can be useful, but only gives an impression of the effect of the drug and not of the overall hemostatic capacity of the patient (161). The effect of DOACs on thrombin generation has been studied quite extensively, with *in vitro* studies as well as with plasma from patients using DOACs.

The *in vitro* studies showed that thrombin generation is hampered by DOACs (162–170). However, the parameters that are affected differ with the kind of DOAC. For example, dabigatran resulted in an increased lag time, while thrombin peak height and ETP remained relatively normal (162–164). On the other hand, presence of FXa inhibitors (apixaban, edoxaban and rivaroxaban) was shown by an increased lag time, but also an increased time to thrombin peak, with additional decreased thrombin peak height and ETP (163–167). Most studies that compare different DOACs, have shown that rivaroxaban has a stronger inhibitory effect on thrombin peak height and ETP compared to apixaban and edoxaban (163, 164). Furthermore, *in vitro* spiking plasma of pediatric and adult patients with edoxaban showed an equal inhibitory effect on thrombin generation among different age groups, except children <2 years of age, who had a stronger inhibition of thrombin generation at the same concentration of edoxaban (169).

Concerning thrombin generation and the use of DOACs, in healthy volunteers taking dabigatran, rivaroxaban and apixaban on different occasions, the same parameters were affected as with *in vitro* measurements (171). Dabigatran only increased lag time, while apixaban and rivaroxaban both inhibited thrombin peak height and ETP (171–174). Further studies showed that apixaban and rivaroxaban have a non-linear inhibitory function for thrombin generation. This indicates that most thrombin generation inhibition occurs at low anti-Xa concentrations (i.e., with a low concentration of DOAC, thrombin generation is still inhibited) (175, 176). Therefore, the authors further investigated how much thrombin was generated 12 h after intake of a DOAC. Thrombin generation was still inhibited at this time point, suggesting that an urgent surgery was not possible when thrombin generation is used as surrogate marker for hemostatic normalization (177). Furthermore, plasma levels of DOACs did not correlate with the extend of thrombin generation inhibition (178, 179). Therefore, thrombin generation measurement to provide an individual thrombin generation profile could be of more importance in a patient presenting with an acute bleeding or in need of urgent surgery than measuring DOAC anti-Xa activity level.

Lastly, several studies have investigated *in vitro* the effects of DOAC reversal therapy. These studies show that a TGA can help to establish correction of thrombin generation after addition of reversal agents (162, 166, 180). This is of major importance because other laboratory assays that do not measure hemostasis as a whole and cannot be used (i.e., anti-Xa assays or factor level activity assays) (161).

#### Antiplatelet therapy

Even though antiplatelet therapy, like aspirin and clopidogrel, do not affect coagulation factors, the effect of these therapies on thrombin generation were studied in PRP as well as PPP. A case-control study showed that patients with CAD followed by PCI who were treated with standard dosage of dual antiplatelet therapy with aspirin and clopidogrel had significant longer time to thrombin peak, decreased thrombin peak height and ETP than controls in PRP (181). Velocity of thrombin generation was most impaired in patients. These differences in thrombin generation parameters were not found in PPP but only in PRP suggesting the importance of platelets (181). In a longitudinal study investigating thrombin generation after ischemic stroke, it was shown that aspirin in combination with dipyridamole significantly decreased thrombin peak height and ETP, while aspirin monotherapy and clopidogrel did not significantly change thrombin generation compared to baseline measured in PPP and not in PRP (182). Another study showed that platelet reactivity, measured with different platelet-activity assays, did not correlate with thrombin generation, measured in PPP (183). These studies show the importance of measurement thrombin generation in PRP, because in PPP it is less sensitive to assess the effects of these drugs.

In a study by de Breet et al., thrombin generation was measured in PPP one and six month after PCI for CAD. Patients were followed for one year to assess bleeding. It appeared that patients with bleeding had a significant lower thrombin peak height, ETP and velocity of thrombin generation at 1 and 6 months after PCI compared to patients without bleeding. Suggesting that performing TGA is possibly to identify patients with clinical relevant risk for bleeding episodes whilst using dual antiplatelet therapy (184).

## Thrombin generation assays to personalize medicine

### Personalized medicine

Personalized medicine is becoming increasingly important in research and clinical practice and aims that “medial decisions, practices, interventions, and/or therapeutic agents are being tailored to the individual patient, based on their predicted response to treatment or risk of disease” (185). In other words, it aims to adjust treatment to each patient individual needs and preferences.

**FIGURE 2:**
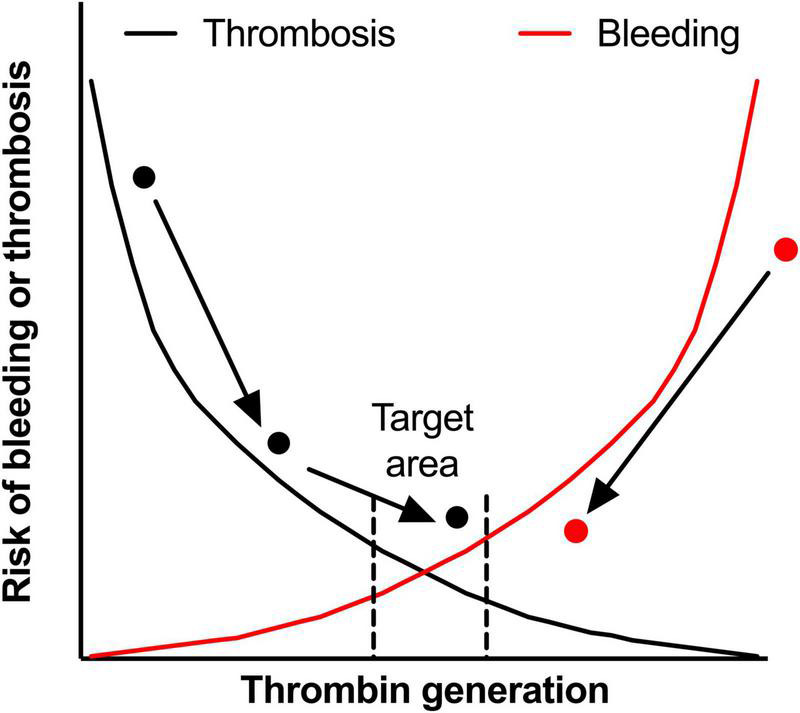
The hemostatic balance. The hemostatic balance is depicted as the risk of thrombosis (black line) and the risk of bleeding (red line), which is dependent on thrombin generation (on the x-axis). In the target area, both the risk of thrombosis and the risk of bleeding are acceptably low, but can still occur in an individual patient in certain circumstances. Two patients are shown in the figure, the black dot represents a patient with a venous thrombo-embolism during an hypercoagulable state. With treatment the hypercoagulable state is diminished, but the patient experiences a second thrombotic event while on adequate anticoagulant therapy. By intensifying anticoagulant treatment, this patient reaches the target area. On the other side of the curve, the red dot represents an hemophilia A patient with a high bleeding rate. After starting prophylactic therapy with factor VIII concentrate, the bleeding phenotype improves, but doesn’t reach the target area. However, for this patient the reduction in bleeding is acceptable, while intensifying treatment could lead to overshoot to a risk of thrombosis. In both patients, monitoring with TGAs could identify the target area better. This could have prevented the second thrombosis in the patient represented by the black dot as the hypercoagulable state was recognized earlier.

The processes of thrombosis and hemostasis are often depicted as a balance or two crossing lines (Figure 2). The lines represent the chance of bleeding or thrombosis, the pathological outcomes, which is probably more represented by thrombin generation in the individual patient. Even within the small target area, patients are still at risk for bleeding and thrombotic events, even though this risk is smaller than at the extremes of the curve. This can for example be seen in a patient with thrombosis (the black dot in Figure 2) due to a hypercoagulable state. With the use of anticoagulation, for example VKA with a target INR of 2–3, the risk of VTE recurrence is lowered, but some patients can still be hypercoagulable while having an INR within the target range (145). Therefore this patient can experience a recurrent VTE and only after intensification of the INR target range to 3–4, the patient’s hemostatic balance is within the referred range. In this example, VKA therapy and monitoring can be seen as a form of personalized medicine, because the number of tablets is dependent of the measured INR value. However, PT-based INR monitoring is highly artificial (due to a high TF concentration) and anticoagulant factors, like APC resistance, are not part of the INR test. Therefore INR is not really monitoring the hemostatic balance, while TGAs are more physiological and are expected to better reflect the patients hemostatic potential.

Because the INR can be sub- or supratherapeutic, with an accessory risk of thrombosis or bleeding, respectively, DOACs were developed. Since DOAC therapy has a standard dosage regimen based on evidence-based medicine, the manufactures advocate that monitoring is not required. However, patients can also be over- or underdosed. This is illustrated by the percentage of bleeding and recurrent thrombotic events in the DOAC trials (6), thereby indicating that for the main population, the dosage of the DOAC is correct, but for a number of patients it still results in either bleeding or thrombosis. TGAs, however, could give an impression of the hemostatic balance of the individual patient. This was illustrated in a case report in which the dosage of rivaroxaban was adapted based on thrombin generation results (186).

In hemophilia treatment, personalized medicine is becoming the standard of care. Prophylactic therapy with factor replacement therapy decreases the bleeding phenotype from a regular and spontaneous bleeder to become a mild bleeder (red dot in Figure 2). By intensifying prophylactic treatment, either by increasing the dosage or shortening the interval, the treatment can be personalized to a situation in which the patient has even less bleeds. However, by intensifying treatment, the chance of thrombotic disease may increase and costs will rise temporarily. Global assays may overcome this as it is expected that these assays better reflect the hemostatic balance. Measurement of thrombin generation can be combined with FVIII activity level and this could be used as the basis for an individualized treatment scheme, with the help of a population based PK-PD model (63, 66). However, this strategy is not yet tested in daily clinical practice.

### Controversies and limitations

Despite the overwhelming amount of evidence in favor of implementing TGAs in thrombotic and bleeding disorders to personalize medicine, some controversies and limitations remain to be addressed. Four important points need to be addressed, of which two are related to the methodology of the TGA and two regarding the monitoring of the hemostatic balance.

First, different kinds of (commercial) platforms exist to determine thrombin generation. In general, all platforms use the same method, but with slightly different concentrations of reagents, which are often undisclosed. This leads to varying amounts of generated thrombin with associated variating normal values. This problem could be targeted by normalizing the TGA parameters with normal pooled plasma (NPP). Even though it is assumed that NPP should approximately contain a normal activity level of all coagulation factors, there is still difference in thrombin generation with different NPP. This was nicely illustrated by a study that investigated the coefficient of variation of three plasmas with different coagulation profiles. Despite using the same TGA, results differed more than the predefined 25% and normalizing the results with the laboratory’s own NPP did not improve variation (187). Therefore, before the TGA can be compared across studies and can be used in daily clinical practice, the methods and materials used should be harmonized.

Second, performance of the TGA is time consuming because of preparation of plasma and the duration of the assay itself. For research purposes this is not a problem, as the TGA is often performed in batches to minimize variation. However, in clinical practice, the need of TGA determination can be urgent, for example, as mentioned in case of bleeding in a HA patient treated with emicizumab, or a patient using a DOAC. The whole blood viscoelastic tests (e.g., ROTEM) can be determined directly and results are available within an hour. Therefore, the determination of the TGA should be faster and possibly applicable as a point-of-care test (POCT). Already progress is being made to develop a POCT TGA which can be utilized at home for monitoring hemophilia patients with a bleed or at the emergency services to screen for coagulation defects in multi-trauma patients.

Thirdly, the target range of TGAs on the hemostatic balance is not known. This means that normal values of a larger healthy population are known, but it is unknown if this range is also the target range to prevent bleeding and thrombosis for an individual patient. Furthermore, each individual has a certain amount of thrombin generation during stable situations. However, during a thrombotic event, thrombin generation can be higher compared to the normal situation due to an intercurrent event which may has caused the thrombosis. The question is to what extent the patient should be treated; to the amount of thrombin generation before the event (if this is known), or to a predefined target based on large evidence based studies. In both cases, the patients treatment is according the principals of personalized medicine. However, only in the first scenario the patient is treated according to its own hemostatic need, which probably prevents bleeding due to the right amount of anticoagulation.

Fourth, the TGA is just one parameter whereas the hemostatic balance is orchestrated by the vessel wall, platelets, coagulation factor and fibrinolysis. The ultimate goal would be a global assay that measures these parameters simultaneously.

## Future perspective and conclusion

Thrombin generation assays can play an important role in the assessment of bleeding and thrombotic diseases, like diagnosis and prognosis of coagulation disorders, both inherited and acquired, as well as monitoring of the treatment of these diseases. However, a number of important items need further attention. The most important is the comparison and standardization of different TGA platforms with uniform reporting of the (local) thrombin generation reference values and normalization to NPP. Furthermore, most of the studies described in this review contain only small numbers of patients and are often monocentric. Larger studies with the use of standardized TGA systems could help for better adaptation of the assay in clinical practice. Also, serial determination of thrombin generation during health and disease (bleeding, thrombosis, and/or hypercoagulable states like cancer) in an individual patient will help understanding the variation in thrombin generation over time. This will give insight in the determination of the target range for treatment of bleeding and thrombotic disorders. Lastly, rapidly available, POCT TGA testing are promising to determine the hemostatic balance of an individual patient with acute hemorrhage due to acquired or congenital bleeding disorders.

In conclusion, TGAs are a versatile tool to measure the coagulation cascade as a whole in bleeding and thrombotic diseases. Since it measures the individual patients hemostatic balance, it can be used to personalize medicine of patients with bleeding disorders, thrombosis and monitoring (anti)hemostatic therapy (see Table 1). Results of recent research show that personalized medicine based on TGAs is most promising for patients with HA. Especially with the emerging non-factor replacement therapies and concomitant usage of therapies, hemostatic monitoring on an individual patient level is essential. The personalization of therapies could ultimately lead to tailoring the treatment of these disorders to needs of the patients without exposing them to unnecessary bleeding or thrombotic risks. However, before utilization in clinical practice, some important hurdles should be taken.

## Author contributions

LV and SR wrote the first draft of the manuscript. DM, SS, and WH critically revised the manuscript. All authors contributed to the article and approved the submitted version.
